# Apicomplexa Cell Cycles: Something Old, Borrowed, Lost, and New

**DOI:** 10.1016/j.pt.2018.07.006

**Published:** 2018-08-02

**Authors:** Michael W. White, Elena S. Suvorova

**Affiliations:** 1 Department of Global Health, College of Public Health, University of South Florida, Tampa, FL 33612, USA

## Abstract

Increased parasite burden is linked to the severity of clinical disease caused by Apicomplexa parasites such as *Toxoplasma gondii, Plasmodium* spp, and *Cryptosporidium*. Pathogenesis of apicomplexan infections is greatly affected by the growth rate of the parasite asexual stages. This review discusses recent advances in deciphering the mitotic structures and cell cycle regulatory factors required by Apicomplexa parasites to replicate. As the molecular details become clearer, it is evident that the highly unconventional cell cycles of these parasites is a blending of many ancient and borrowed elements, which were then adapted to enable apicomplexan proliferation in a wide variety of different animal hosts.

## Are Apicomplexan Parasites So Unique?

Papers and reviews describing asexual cell division of apicomplexans often use many wonder type adjectives such as unusual, peculiar, unprecedented, and remarkable (we are guilty as charged). The problem with our collective habit is that it effectively treats these protozoans as aliens on Earth and inevitably misses compelling biological backstories and what is truly unique about these important pathogens.

The Old English Rhyme,

‘Something old,

Something new,

Something borrowed, and

Something blue’,

that describes the essential ingredients of marriage does a much better job of portraying the melding of processes that together produce apicomplexan biology (using an alternative meaning of blue – ‘lost’). In this review, we focus on recent advances in understanding the structural and molecular basis of cell division in the model apicomplexan, *T. gondii*, placing where possible new discoveries in an evolutionary context. Our strategy for evaluating evolutionary contexts is to assume new is rare, look for old first in genomics and cell biological evidence. We then assess whether the trait is borrowed from algae or plants. We keep in mind that often pathway topology may be conserved while individual protein elements are not, which especially applies to cell cycle regulatory networks [1].

## Understanding the Evolution of the Apicomplexa Phylum

**Figure 1.**
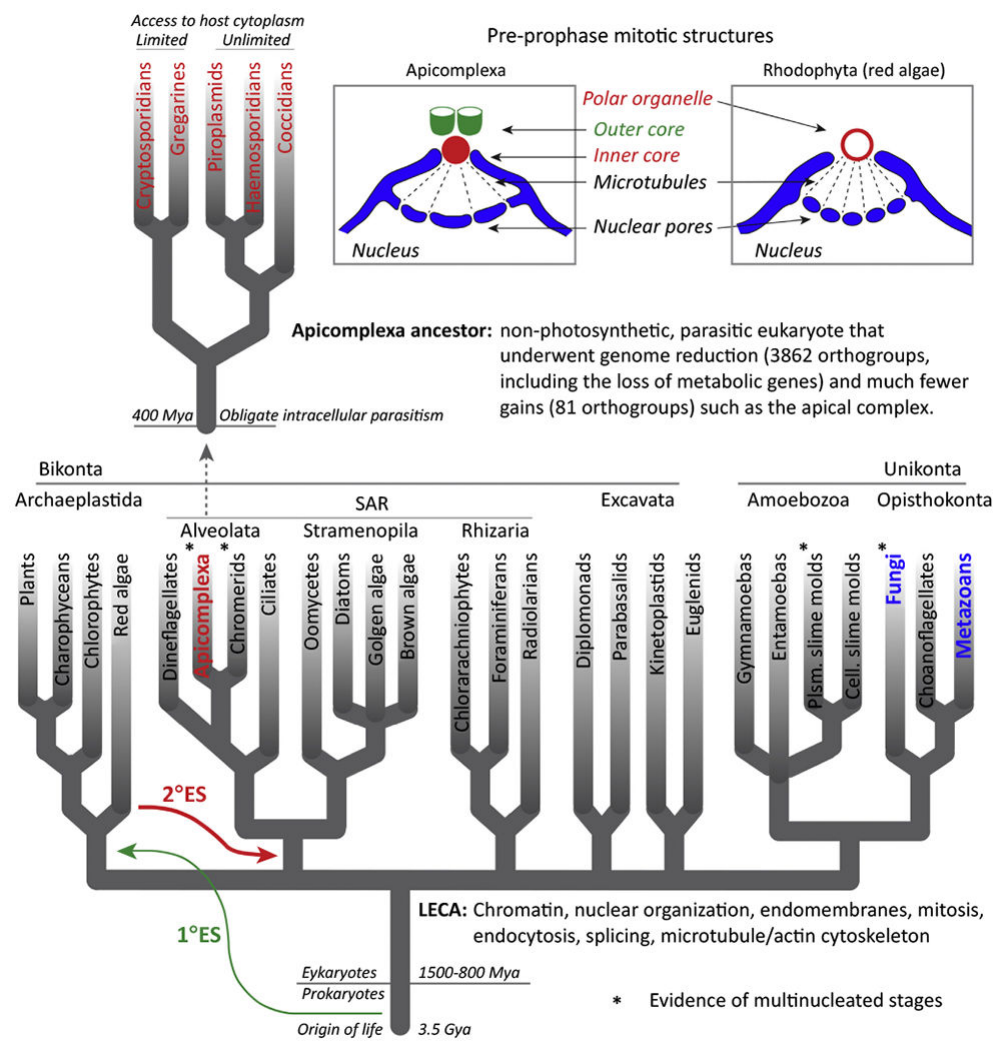
Evolutionary History of Apicomplexa. (A) Phylogenetic relationships of major eukaryotic groups are shown [2,73]. Two bikont groups acquired piastids, Archaeplastida by primary endosymbiosis (1°ES, green arrow) of ancestral cyanobacterium, and Chromalveolates by secondary endosymbiosis (2°ES, red arrow) of red algae [73]. Please note that schematics indicate the earliest predicted time of the secondary endosymbiosis, while the number and order of tertiary events are still a topic of discussion (for more details see recent reviews [74,75]). Common nuclear functions of modern eukaryotes were present in the last eukaryotic common ancestor (LECA) [5,73]. Adoption of multinuclear replication, such as schizogony, occurred in the proto-Apicomplexa ancestor (asterisks) prior to the switch to obligate parasitism [7,8]. Genetic reduction is the major feature of the Apicomplexa ancestor switch to intracellular parasitism [3]. Well-studied conventional eukaryotic cell cycles that obey ‘once only’ rule and complete cytokinesis following chromosome segregation are indicated in blue, while eukaryotic model systems (e.g., *T. gondli*), where unconventional cell cycles are utilized, are indicated in red. (B) Red alga and Apicomplexa spindle pole complexes have many morphogenetic similarities indicating a potential common evolutionary history. Schematic shows pre-prophase mitotic structures of *T. gondii* (Apicomplexa) and *Apoglossum ruscifolium* (Rhodophyta). The *Toxoplasma* centrocone is a composed image of the numerous electron microscopy studies. Note that the inner core was only detected by immunofluorescent microscopy [13,27]. The mitotic structure of the red algae was sketched from images 2 and 3 of the study by Dave and Godward, 1982 [30]. During mitosis, nuclear membrane protrusions (centrocone in the Apicomplexa) are initiated at cytoplasmic protein complexes (inner core versus polar organelle). Local fenestra then form at the nuclear protrusions (semi-closed mitosis) through which spindle microtubules extend into the nucleus and attach to segregating sister chromatids.

At the dawn of the Apicomplexa lineage divergence, several hundred million years had likely passed since the emergence of the last common ancestor of eukaryote, **LECA** (see Glossary) [2] (Figure 1A and Box 1). The ancestor of apicomplexans is thought to be similar to the present-day free-living phototrophic algae [3] and is dated to the beginning or later than the emergence of the Archaeplastida plant lineages based on the plastid organelle acquired by secondary endosymbiosis of a red alga [4,5] (Figure 1A). The driving force of subsequent Apicomplexa lineage divergence is the complete switch to a parasitic life style, and this led to significant reductions of genetic content [3]. It is estimated that >4000 orthologous genes in the proto-apicomplexan ancestor were lost in some apicomplexan lineages. The proteins lost [3] include flagella proteins and those needed for photosynthesis and some scavenging processes. In other words, if it were needed for a free-living life style it might not be present in extant apicomplexans. As predictable as gene losses are, the small gains in gene function also make sense. Secretory proteins needed to invade and survive within a host, and a new motility apparatus adapted to a multilayered cytoskeleton, highlight the functions gained during Apicomplexa phylum evolution.

## Ancestral Innovations Provide the Structural Foundation of Cell Division in *Toxoplasma* Tachyzoites

**Figure 2.**
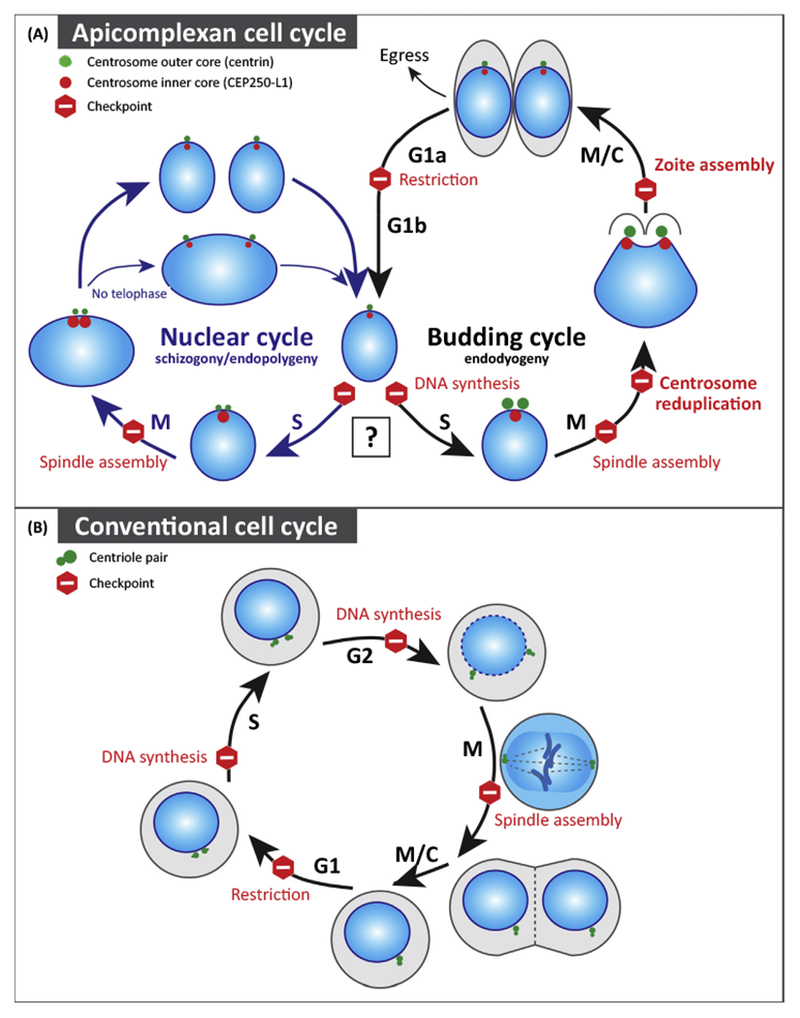
Schematics of the Apicomplexan (A) Compared To Conventional (B) Cell Cycles. In the conventional cell cycle, chromosome replication (S) and segregation (M, mitosis) phases are separated by two growth phases, G1 and G2. The diagram shows major checkpoints regulating somatic cell division that ensure successful completion of key events such as sufficient cell growth, complete replication of chromosomes, and the secure attachment of chromosomes to the spindle poles (see details in Box 2). The conventional cell cycle is governed by the ‘once only’ rule of chromosome replication that is functionally linked to centrosome duplication shown in the diagram as a centriolar pair. By contrast, the apicomplexan cell cycle is more complex and has different regulatory points. At the G1/S phase transition, parasites may choose one of two different chromosome cycles. They can enter either a nuclear cycle (blue arrows) where chromosome replication is not accompanied by budding or a budding cycle (black arrows) where chromosome replication and segregation is synchronized with the assembly of the daughter buds. Although signals that direct parasites into each type of cycle are largely unknown, recent studies of *Toxoplasma gondii* tachyzoites demonstrated that a bipartite centrosome plays an important role in the decision. Results indicate that an active outer core (green) favors the budding cycle route. Many apicomplexan parasites utilize the nuclear cycle (e.g., *Plasmodium* spp. schizogony) to significantly increase the number of the parasite progeny from a single infection event, including *Toxoplasma* merozoites (endopolygeny) in the cat life cycle. Note that mitosis of the nuclear cycle of the *Sarcocystis neurona* endopolygeny lacks telophase. Thus, *S. neurona* nucleus division occurs only in the budding cycle. Additional regulatory points evolved to regulate these complex parasite cell cycles (bolded letters), including reduplication of the centrosome in mitosis and assembly of the daughter bud cytoskeleton.

All apicomplexan life cycles have two basic objectives, to produce sufficient progeny to perpetuate existence and to form the specialized stages needed for successful host transmission. Asexual stages are responsible for meeting the biotic mass requirements. Thus, replication terminology is based on where new parasites are formed; when daughter parasites are formed internally the process is called **endodyogeny**/**endopolygeny**, while budding from the mother plasmalemma is called **schizogony** [6]. The binary replication of the *Toxoplasma* tachyzoite is the simplest proliferative scale as most apicomplexan cell divisions produce more than two nuclei with concerted budding accompanying the last nuclear reduplication (Figure 2). Multinuclear replication is not a parasitic trait, because many free-living eukaryotes utilize this strategy, including the nearest free-living relatives of the Apicomplexa, chromerids [7,8]. The switch to a parasitic lifestyle may be responsible for the extraordinary scales and the near universal adoption of this type of replication in apicomplexan parasites.

**Figure 3.**
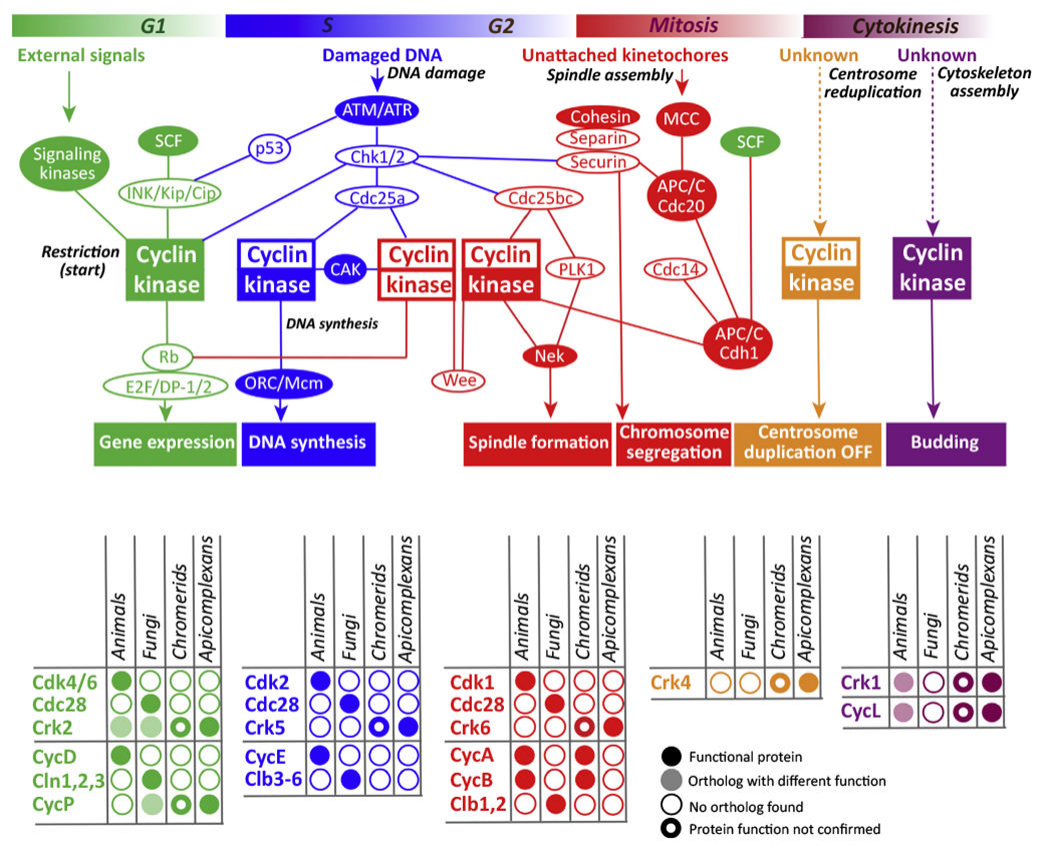
Schematics of the Prototypical Eukaryotic Cell Cycle and Key Regulatory Mechanisms. To highlight differences in the number and composition of controls, we superimposed the recent findings from apicomplexan *Toxoplasma gondii* onto the conventional cell cycle model of such extensively studied opisthokont organisms as animals and fungi. The schematic shows only key regulators of the complex network governing progression through specific cell cycle phases (color bars on the top). *Toxoplasma* factors orthologous to opisthokont regulators identified by pBLAST search as well as conserved processes are shown as filled shapes (‘old’). Missing factors indicated as open shapes (‘lost’) would be candidates for analogs (‘new’) or adapted factors (‘borrowed’). Identifiers of the central kinases and cyclins are shown in the corresponding tables below. Opisthokont regulators are listed along with factors detected in the apicomplexan ancestors, Chromerids (*Chromera velia*). The Apicomplexa phylum is represented by the well-studied *T. gondii* tachyzoite model. Our analysis revealed that novel apicomplexan central kinase/cyclin complexes also lack expected immediate regulators of opisthokont eukaryotes such as G1 Cdk inhibitors (INK, Kip, Cip), Cdc25 phosphatases, and Wee1 kinase [62], consistent with cell cycle mechanisms evolving as a unit. Apicomplexan mitotic Crks are missing cognitive cyclins. Note that, unlike opisthokont Cdks, multiple apicomplexan Crks are nonredundant, indicating that the true master regulator of the apicomplexan cell cycle is not yet identified.

Cell division in *Toxoplasma* tachyzoites is founded on a number of ancestral mechanisms, especially those processes for copying (S phase) and segregating (mitosis) chromosomes (Figure 2). Present apicomplexans possess the core DNA synthetic machinery conserved across eukaryote domains, including all subunits of the MCM helicase complex and functionally specialized DNA polymerases [9,10] (Figure 3 and Box 2). Key factors that direct the DNA synthetic machinery to specific initiation sites in the chromosomes are also present [9]. The sequences of the origins of DNA replication (ori) that have been recently mapped in *Plasmodium falciparum* appear to be similar to those in yeast [11]. There are differences noted in some of these DNA synthetic mechanisms, mostly as the result of gene loss [9,10]. The apicomplexans have conserved the kinetochore proteins and the histone CenH3 that specifically binds centromeres [12]. Thus, chromosome spindle attachment mechanisms in *Toxoplasma* also belong in the ‘old’ category. Tachyzoite intranuclear spindles are typically short, but can span the 1–2 μm nucleus [13,14]. Centromeres are clustered and bound to the nuclear membrane near the spindle pole in all cell cycle phases except mitosis (spindle forms in mitosis only) [15,16]. This structural arrangement likely involves apicomplexan contributions. However, as a strategy, chromosome clustering near the spindle pole throughout the cell cycle is observed in many unicellular eukaryotes [17], and this feature permits the unikont fission yeast to complete nuclear division in the absence of a spindle [18].

Chromosome segregation using a bipolar microtubule spindle is an original LECA innovation that has been modified many times during eukaryotic evolution [19,20]. A number of recent studies of *Toxoplasma* mitosis have uncovered such a mixture of ancient and newer innovations operating in tachyzoite cell division [13,15,21]. Like all apicomplexans, tachyzoite chromosomes are segregated within the nucleus, although the terminology used to describe mitosis in these parasites can be confusing. High-resolution images of coccidian asexual stages, including *Toxoplasma* tachyzoites, show that the nuclear membrane is open to the cytoplasm at the spindle pole during mitosis [22–26] (Figure 1B) and then closes during nuclear division [22]. This arrangement was recently confirmed in studies of the spindle binding protein, EB1, which showed that deletion of the EB1 nuclear localization signal did not prevent bipolar spindle localization [14]. It seems clear from these data that *Toxoplasma* tachyzoites divide by **semi-closed mitosis**, which is a process widely used in all major eukaryotic groups and was very likely the form of mitosis used by the LECA [19]. The advantages of semi-closed mitosis for apicomplexan replication are several; this process eliminates the need to transport spindle components into the nucleus, it permits open communication with the cytoplasmic microtubule-organizing center (**MTOC**) responsible for budding, and it provides an easy method to coordinate multinuclear reduplication while preserving the safety of chromosome segregation within an intact nucleus.

At the other end of the tachyzoite spindle from the centromere is the MTOC embedded in the nuclear membrane called the **centrocone** that is present in many, if not all, apicomplexan parasites (Figure 1B). We have recently discovered a cytoplasmic protein complex (inner core) that is tightly aligned with the centrocone of *Toxoplasma* tachyzoites [13]. The inner core is part of a larger **bipartite centrosome** in *Toxoplasma* that also has an outer core [13]. The inner and outer cores of the tachyzoite centrosome function and duplicate independently and may have distinct evolutionary history. The inner core is copied after the outer core in S phase (Figure 2), and contains orthologs of the coiled-coiled centrosomal proteins of higher eukaryotes, while lacking centrin [13,27]. Functionally, the inner core is required for chromosome replication in the tachyzoite [13,28], and in genetic mutants that over-duplicate the inner core the centrocone is also over duplicated [13]. These results indicate that the inner core and centrocone constitute a complete spindle pole complex in these parasites. There is no clear evidence that this spindle pole complex has a centriole, and thus, it may be ancestrally related to the spindle pole plaques of other Apicomplexa that also lack centrioles, such as *P. falciparum* merozoites [29]. During peak mitosis the parasite centrocone structure locally separates the nuclear membrane, as can be seen in many transmission electron microscopy images of the centrocone [22–26]. The centrocone nuclear protrusion is highly reminiscent of the spindle pole of red algae, which also develops polar nuclear protuberances that lack centrioles (red algae also lack flagella, see Figure 1B) [30]. The red alga spindle pole is thought to be formed by clustering nuclear pores and concentrating proteins within the localized nuclear envelope. Similar to the tachyzoite spindle pole, the red alga spindle pole initiates with the formation of protein structures on the cytoplasmic face of the nascent polar protrusion and involves temporary breaks in the nuclear membrane during mitosis. The polar ring above the nuclear protrusion in red algae may be analogous to the *Toxoplasma* tachyzoite inner core or was replaced by the outer core of the bipartite centrosome. The structural similarity between the red alga and coccidian spindle poles was noted more than 35 years ago [30], long before a red alga ancestor was implicated as the source of the plastid in the Apicomplexa. If these structures are ancestrally related, it places the tachyzoite spindle pole complex into the category of ‘borrowed’ from the secondary endo-symbiotic event. Just seven *Toxoplasma* proteins have been currently localized to the tachyzoite spindle pole complex (**CEP**250, CEP250-L1, CEP530, ECR1, **Crk**5, MORN1, and EB1) not including microtubules [13,14,27]. However, we expect to discover additional apicomplexan-specific contributions to these structures. Altogether, the conclusions that can be drawn from comparative genomics [31] and morphogenesis of DNA synthetic and karyokinesis-related processes in the *Toxoplasma* tachyzoite and other apicomplexans are that these mechanisms long predate these parasites and are mostly in the ‘old’ category.

Invasion mechanisms represent some of the newest innovations in Apicomplexa biology [3]. This includes a novel method of myosin-actin based motility (glideosome) that is embedded into a layered cytoskeleton comprised of an apical complex, subpellicular microtubules, and cortical alveoli (inner membrane complex). The molecular details and the process of assembling the apicomplexan daughter cytoskeleton have been recently reviewed [32,33]. Biosynthesis of the daughter cytoskeleton is the major activity of apicomplexan cytokinesis, which is suspended in multinuclear replication until the last round of chromosome replication. We have discovered that initiating the new daughter cytoskeleton in *Toxoplasma* tachyzoites is the function of the outer core of the bipartite centrosome [13] that possesses a centriole, which duplicates and orients in an unusual parallel arrangement [16] as parasites enter S phase [13]. Centrin and its binding protein, Sfi-1, exclusively bind the outer core as does Aurora and Nek family kinases [13,34]. The first markers of daughter budding [35], the inner membrane complex protein 15 (**IMC**15) and the protein trafficking GTPase (Rab11b), can be detected colocalized near the duplicated outer core. Progressive growth of the tachyzoite daughter bud involves the incorporation of many proteins, including MORN1 [6], and the polymerization of striated fibers (SFA) that originate near the outer core appears to drive bud extension [21]. Importantly, genetic disruption of the outer core prevents the initiation of parasite budding without effecting spindle pole duplication (inner core and centrocone) or karyokinesis [13]. The presence of centrioles, centrin, Rab11b, and SFA fibers emanating from the outer core is a feature similar to the flagellar rootlet system or basal body cage of the green alga *Chlamydomonas reinhardtii* [36]. The function of the rootlet system is to define flagella position as well as cellular polarization, which is a newly appreciated role for related centrosome and basal body structures of higher eukaryotes [37]. Thus, it is clear that the evolutionary origin of the outer core is founded on the history of flagella motility and is distinct from the proposed red algae origin of the inner core/centrocone spindle complex. Importantly, apicomplexans possessing these structures have adapted two distinct MTOC complexes in order to independently control karyokinesis and cytokinesis. This new centrosome innovation should enable these parasites to switch between nuclear and budding cycles such that daughter parasites can synchronously release from the infected host cell (Figure 2).

## Overview of Apicomplexan Cell Cycle Regulation

Like the structural components of cell division in the previous section, the regulation of the apicomplexan cell cycle reflects the blending of ancient and adapted mechanisms. Despite a variety of the morphological patterns among apicomplexan parasites, there are clearly distinguishable cell cycle phases changing in precise order (Figure 2) [38]. For example, the *Toxoplasma* tachyzoite cell cycle begins with the gap period (G1 phase) that is first devoted to the biosynthesis of protein and RNA components (G1a) followed by a switch to the component needs of DNA synthesis in the second half of G1 (G1b) [39]. This is the same general biosynthetic order of G1 phase in our own cells. Like other eukaryotes, *Toxoplasma* chromosomes are replicated in S phase and segregated in mitosis. We do not know if a G2 period is short or absent [38] in these parasites, however, the lack of a discernable G2 period is not uncommon in unicellular eukaryotes, including other apicomplexans [40,41].

We and others have shown that the function of certain proteins is linked to a specific tachyzoite cell cycle stage [13,34,42–47], and the ability to reversibly arrest the *Toxoplasma* tachyzoite cell cycle [48] operationally demonstrates that the conserved rule of the checkpoint regulation in apicomplexans is in place. The general topology of cell cycle regulation is conserved too. At critical cell cycle transitions, similar signals are generated and recognized by identifiable paneukaryotic orthologs, and a similar outcome is produced upon checkpoint satisfaction (‘old’ Figure 3, filled shapes). However, central regulators and immediate effectors are changed due to initial loss (‘lost’) followed by re-tooling during genome expansion stages (‘new’) (Figure 3, open shapes). Apicomplexans likely ‘borrowed’ via secondary symbiosis a family of plant-specific **AP2** transcriptional factors [49] that are anticipated to fulfill the role of missing **Cdk** effectors driving cell cycle progression in higher eukaryotes. Notably, truly new and unique regulatory mechanisms are associated with such specialized structures as the bipartite centrosome [13] and the cytoskeletal layers (‘new’) [32] that are functionally linked to the separate nuclear and **budding cycles**.

Based on comparative genomics of extant eukaryotes, it is thought that the primordial eukaryotic cell cycle was driven by activation of a Cdk/cyclin complex that triggered DNA replication and mitotic spindle assembly. This was then followed by APC/C-induced cyclin destruction that triggered removal of the sister chromatid tethers, chromosome segregation in anaphase, and formation of the DNA prereplication complexes for the next division cycle [50–52]. Phylogenetic evidence gathered from various eukaryotic groups indicates that the LECA had a rather complex cell cycle regulation based on a single Cdk1 type kinase that paired with multiple cyclins A-, B-, D-, and E-type, and was controlled by the transcription factor **E2F**, retinoblastoma protein (**Rb**), and APC/C family proteins [53]. Some eukaryotic groups retained this basic regulatory network, while the others significantly increased its complexity. For example, modern mammalian Cdk4/6 kinase activated with D-type cyclins controls G1 phase progression; Cdk2 and E and A-type cyclins promote S phase; Cdk1 activated by cyclins A and B regulates mitosis (Figure 3) [54,55]. Recent evaluation of apicomplexan genomes and the ancestrally related chromerids confirm that the apicomplexan ancestor possessed the basic cell cycle regulatory machinery of the LECA [3,42,53,56].

## Control of the Apicomplexan G1 Phase

To provide differential control of periodic and constitutive synthesis, the eukaryotic cell cycle alternates between growth and DNA replication/segregation phases, always obeying the rule that cell size dictates the time and scale of replication [52,57], Apicomplexans appear to follow this old rule where the number of chromosome duplications **(nuclear cycles)** [13,28], and ultimately the scale of progeny production, are predetermined by the time parasites spend in the G1 growth phase. For example, *T. gondii* tachyzoites and *P. falciparum* merozoites produce 2 versus 32 progeny, respectively, after G1 phases of ~3 versus 12 h. At the extreme end of Apicomplexa replication, is the ~6 day G1 phase of the *Eimeria bovis* sporozoite that produces thousands of daughter parasites. Central G1 regulatory machinery of apicomplexans is an exemplary mixture of ancestral and novel features (Box 2). Phylogenetic analysis revealed that canonical G1 Cdk/Cyc pairs of yeast and higher eukaryotes are absent from nearly all the branches of Apicomplexa parasites (Figure 3) [42]. Instead, *T. gondii* tachyzoite G1 phase is regulated by Cdk5 (PHO85)-related kinase TgCrk2, which pairs with P/U-type cyclin. Such an unusual alliance likely controls the transition through the restriction point called START in yeast (*T. gondii* – G1a/G1b stages, *Plasmodium* spp. – ring/early trophozoite stages), because the loss of either regulator blocks *T. gondii* tachyzoite growth in the G1 phase [42]. The dominating expression of P/U cyclin in the cytoplasm of *T. gondii* and *P. falciparum* [42,56] correlates with the function of ancestral P/U-cyclins to sense environmental changes during the G1 period [58]. The presence of P/U cyclins and Cdk5-related kinases in modern plants [59], fungi [60], and kinetoplastids [61] argues that this complex may have existed in the LECA and regulated the ancient G1 phase, and thus, potentially placing this mechanism into the ‘old’ category.

The apicomplexan G1 phase is also a perfect example of preserving the network structure rather than individual components [1,62]. In higher eukaryotes, the canonical G1 Cdk/Cyc pair activates specific transcriptional response via the E2F/**DP-1** pathway counterbalanced by the activity of tumor suppressor Rb protein and is under control of the INK/Kip/Cip family of Cdk inhibitors (Figure 3). Obligatory intracellular apicomplexans such as *T. gondii* and *Plasmodium* spp. have lost many critical components of this network (Figure 3), although immediate effectors of the E2F/DP-1 families and Rb factors are present in the early-branched apicomplexans with distinctive semi-intracellular stages (Figure 1A; cryptosporidians and gregarines) [53] (eupathDB). Instead, the majority of apicomplexans possess a large group of AP2 transcriptional factors that were ‘borrowed’ from the red algae during secondary symbiosis [63] (Box 2). Therefore, it is reasonable to hypothesize that some of these AP2 factors may transduce a signal from the activated Crk2/P-cyclin complex and trigger G1 gene expression analogous to the E2F/DP-1 signaling pathway. It is possible that, similar to SBF inheritance in yeast, AP2 factors may have replaced the E2F/DP-1 mechanism via a hybrid intermediate, because *Cryptosporidium* spp. encode transcription factors of both AP2 and E2F families [53]. Further studies will be needed to validate which group of transcription factors is functional in *Cryptosporidium* cell cycles. The change in the identity of G1 critical players may also explain the ‘loss’ of recognizable orthologs of the INK/Kip/Cip family of Cdk inhibitors and Rb analogs (‘new’ or re-tooled factors) which are yet to be found in apicomplexan parasites.

## Violation of the ‘Once Only’ Replication in Apicomplexans

Since modern species of fungi [64,65], amoeba (slime molds) [66], and alveolates *(Chromera velia)* [67] have binary and multinucleated life stages (Figure 1A, marked with an asterisk), one can assume that apicomplexan schizogony/merogony is an ‘old’ inherited feature, and the basic regulatory machinery should have been passed along with the structures. Unlike binary replication, polyploid division has additional nuclear cycles of repeated chromosome replication/segregation with suppressed cytokinesis (Figure 2) [13,16]. Multiple nuclear cycles clearly violate the ‘once only’ mechanism of the eukaryotic DNA replication that was enforced by separation of S and M phases at the time when chromosome condensation incompatible with DNA replication had evolved [52,57]. Thus, the ability to produce large numbers of apicomplexan progeny needed to survive in the host maybe the high price paid for potential unfaithful DNA replication during overlapping S/M phases of polyploid cycles.

The principle mechanism of ‘once only’ control in the eukaryotic cell cycle is irreversible because it is based on proteolytic degradation of the key regulators. For example, cyclical expression of cohesins holding sister chromatids together is the result of **APC/C**-induced degradation during metaphase-to-anaphase transition followed by a new cohesin synthesis in S phase [68]. The presence of cohesins in apicomplexan genomes (eupathDB) suggests that the mechanism holding chromatids together is ‘old’, while relicensing of DNA replication may have several ‘new’ features. There are no identifiable orthologs of separins or securins (‘lost’) in obligatory intracellular apicomplexans (Figure 3), suggesting that these parasites have evolved new regulatory links and equipped them with either novel or re-tooled factors. Among putative candidates is the OTU family of deubiqitinases that have the ability to turn off the relicensing switch in *T. gondii* dyogenic division [69].

The apparent violation of the ‘once only’ rule raises a number of complications. This rule should be incompatible with asynchronous S and M phases, because chromosomes in a prereplicative state have to coexist with postreplicative chromosomes. The mechanisms allowing apicomplexans to distinguish these two chromosomal states in the same mother parasite are currently unknown. One of the logical suggestions is implementation of local controls [16]. We have recently discovered that *T. gondii* tachyzoites confine key regulatory factors to the centrocone [10], which is the perinuclear compartment of the spindle pole complex described in the previous section. It has been suggested that initiation of chromosome replication in *T. gondii* tachyzoites is regulated by Apicomplexa-specific kinase Crk5 [10] ergo ‘new’ (Box 2). Although the timing of TgCrk5 expression suggests a mechanism similar to that in other eukaryotes, where origins of replications are preloaded in mitosis of the previous cycle [70] (Figure 3), *Toxoplasma* may have adapted this mechanism to the needs of apicomplexan biology by holding TgCrk5 and its cofactor TgECR1 in the centrocone until the spindle break in the anaphase transition. *Plasmodium* Crk5 is similarly localized in dot-like structures reminiscent of the centrocone [71], and smaller progeny in PfCrk5-deficient parasites may be the result of deficient licensing of DNA replication. However, there is a fundamental difference between *Toxoplasma* and *Plasmodium* Crk5 that may explain how relicensing is permitted in *P. falciparum* schizogony and suppressed in *T. gondii* endodyogeny. Unlike PfCrk5, that is permanently restricted to the nuclear envelope, TgCrk5 has a full nuclear expression stage in post-anaphase, likely switching from local to global control of the licensing of DNA replication. A closer examination of *P. falciparum* merozoites might find a similar mobilization of PfCrk5 to the cytoplasm only in the last nuclear cycle.

Relicensed DNA replication requires reduplication of MTOC (centrosome or spindle-pole plaque) in preparation for segregation of reduplicated chromosomes. Although mechanisms that coordinate these processes in schizogony are currently unknown, it is plausible to suggest that the progression of asynchronous nuclear cycles can be stopped by TgCrk4 related kinase, which is required to suppress centrosome reduplication at the end of mitosis in *T. gondii* binary division [42]. Alternatively, cytoplasmic TgCrk4 could simply coevolve with the bipartite centrosome described in the section above as the means to coordinate duplication of the outer core [13]. In any case, from a structural and regulatory point of view, the centrosome reduplication checkpoint is a truly ‘new’ invention. Interestingly, novel relicensing TgCrk4 kinase likely works in coordination with another novel Crk6 kinase (Crk4 in *P. falciparum)* [43] that in apicomplexans is expected to regulate an ancient checkpoint of the eukaryotic cell cycle – the metaphase-to-anaphase transition or spindle assembly checkpoint (SAC). Many key components of this ‘old’ mechanism have identifiable orthologs in apicomplexans (MCC, APC/C, Cdc20, Cdh1, cohesins, Figure 3) [72] that may be under the control of ‘new’ nuclear Crk6 kinase (Box 2). To connect old machinery and ‘new’ protein kinases, apicomplexans appear to have established new regulatory links where dynamic expression and lack of identifiable cyclin partners are new integral features (i.e., Crk4, Crk5, Crk6).

## Concluding Remarks

Modern apicomplexans carry in their genomes a history of the phylum evolution. Original LECA features were complemented by acquisition of a plastid (and maybe cilia/flagella) followed by dramatic reduction of the genome and specialization as an obligate intracellular parasite. Likewise, mechanics of the cell cycle and its regulation go side-by-side with morphological changes and adaptations of the apicomplexan parasites: every cell cycle stage is composed of the ancient, novel, and adopted features (see Outstanding Questions). What the amazing images of dividing Apicomplexa parasites reveal is a unique relationship between karyokinesis and cytokinesis that likely arose out of two evolutionary lineages, the original eukaryotic unicellular predator and its red alga symbiont. Apicomplexans melded these backgrounds to develop multiple MTOCs, novel mitotic structures, and a modified regulatory network that switches between two distinct chromosome cycles. It is clear from multiple genetic studies that disrupting these new cell cycle mechanisms leads to fatal outcomes, which has yet to be fully exploited therapeutically to treat the diseases caused by these pathogens. Unfortunately, there is a big knowledge gap in biology of non-unikont organisms mostly due to the limited number of the available models. Sequencing of additional bikont unicellular genomes will be a first step to refine the evolutionary history of apicomplexans (and other non-unikonts).

## Highlights

Do Apicomplexa cell cycles represent a new paradigm? Compared to other eukaryotes (yeast and mammals), images of dividing Apicomplexa parasites might suggest so.

Yet, new comparative genomics and morphogenic research tells a different story of ancient and borrowed features adapted to yield new parasites.

The *T. gondii* bipartite centrosome with functionally independent inner and outer cores provides a new molecular logic for flexible nuclear cycles.

Aurora, Nek, Crk kinases and E3 ligase tethered to *T. gondii* mitotic structures provides evidence for local nuclear control, which is needed to explain the apparent violation of ‘copy once rules’ in multinuclear replication.

If there is a new paradigm it is the suspension of concerted budding until the last nuclear cycle. Here there are new data for greater Crk complexity that has been freed from requiring a cyclin partner.

## Box 1. The Surprising Complexity of the LECA

The first organism on Earth was a prokaryote that emerged ~3.5 billion years ago, and this type of cell dominated life for several billion years [73]. Eukaryotes were thought to have appeared within the marine environment 800–1500 million years ago [2,73,76], and it is proposed that dozens of new innovations in the last common ancestor of eukaryotes (LECA) were required to establish the eukaryotic lineage [73,76]. First innovations required other innovations leading to yet more innovations and so on. For example, the packaging of eukaryotic chromosomes (histone-nucleosomes) into chromatin eliminated the size restrictions that have governed bacterial chromosomes throughout their history [77] and permitted the nascent eukaryote genome to quickly expand with the addition of hundreds of new genes and paralogs [63]. Nuclear compartmentalization of chromosomes required a new type of replication called mitosis, and the separation of gene transcription and translation led to new processes for producing mature mRNA and ribosomes. Nuclear developments also required the development of pores and new transport mechanisms. The take-home message of this brief summary is that the first eukaryote cell was likely more complex than some modern eukaryotes and not nearly as primitive as commonly assumed. This new conceptual view of the LECA is extensively discussed in two recent reviews [73,76].

## Box 2. Conventional versus Apicomplexan Cell Cycle

### G1 Phase

The apicomplexan G1 phase may be initiated by sensing and transducing environmental signals via the activity of signaling kinases [78,79], which triggers an ordered biosynthetic cascade [39,80]. The G1 checkpoint mechanism is an atypical G1 kinase Crk2 in complexwith a P/U-cyclin. Typical ofeukaryoticG1 topology, low Crk2 activity in *Toxoplasma gondii* associates with G1 arrest, which is likely equivalent to eukaryotic Restriction or START-like checkpoints (‘old’). Traditional immediate G1 effectors of E2F/DP-1/Rb (or SBF/Whi5) families are ‘lost’ in many Apicomplexa genomes and are possibly replaced (‘borrowed’) with the ApiAP2 family of transcriptional factors that were acquired from the red alga symbiont.

### S Phase

Apicomplexan S phase has a DNA synthesis checkpoint that controls two processes: DNA damage is regulated by the inherited (‘old’) ATM kinase [81], while licensing of DNA replication is likely regulated by the ‘new’ kinase Crk5 [10]. DNA-replication machinery operated by novel Crk5 (ORC/Mcm) is ‘old’ and nearly complete [9]. Surprisingly, the oldest ancestral checkpoint kinase Chk1 that connects G1, S phase and mitosis is missing in apicomplexans and chromerids along with factors from the Chk1 network, which supports the concept that coevolved complexes are preserved or lost as a unit [62]. Please note that conventional cell cycle has an additional DNA damage/replication checkpoint in the G2 phase that is likely lost in apicomplexans due to the absence of a detectable G2 period and associated regulators, such as Cdc25a phosphatase (see Figure 3 in main text).

### Mitosis

Mitosis in apicomplexan cell division has retained many ‘old’ components of the primordial cell cycle. Duplication and segregation of MTOC (centrosome or centrosomal plaque) that establishes spindle poles for chromosome segregation is similarly regulated by serine/threonine protein kinases of Nek and Aurora families [9,13,34,82,83], while polo-like kinases (PLKs) are ‘lost’ in apicomplexans and ancestral chromerids. Similar to canonical spindle-assembly checkpoint (SAC), apicomplexan parasites regulate metaphase-to-anaphase transition. While many components of the SAC network are preserved in apicomplexans (‘old’: MCC – mitotic checkpoint complex, APC/C – anaphase-promoting complex/cyclosome) [72], the novel (‘new’) Crk6 [42,43] controls exit from mitosis.

### Cytokinesis

The most outstanding feature of apicomplexan division is the ability to postpone cytokinesis during nuclear cycles (repeats of the S/M phases). This innovation may explain the short or absent G2 phase in Apicomplexa as well as the new centrosome/spindle complex relationship that independently controls karyokinesis and cytokinesis. Not surprisingly, this novel regulatory point is under the control of the ‘new’ Crk4 kinase in *T. gondii* [42]. Finally, assembly of the multilayered cytoskeleton unique for alveolates is regulated by a re-tooled (‘new’) Crk1/CycL complex distantly related to the eukaryotic Cdk11/CycL complex of transcriptional regulators [42].

## Outstanding Questions

Are karyokinetic *T. gondii* mitotic structures of red alga origin? Specifically, can we find molecular evidence that components of the spindle pole complex were inherited via secondary endosymbiosis? If mitotic structures are of red alga origin, it raises an additional question about the origin of the apicomplexan nucleus. Is the foundation of the modern apicomplexan nucleus of the predator or the symbiont? Comprehensive phylogenetic analysis of nuclear proteins could shed light on this question.

Do apicomplexans with limited access to the host cytoplasm (*Cryptosporidium* spp. and gregarines) represent a transition stage to the elimination of conventional cell cycle regulatory machinery of the host? And why was this needed?

What signals are generated to activate a switch from asynchronous nuclear cycles to the final synchronized budding cycle during polygenic cell division?

Does the fact that five apicomplexan Crks provide independent control of *T. gondii* tachyzoite replication mean that the true master regulator of the apicomplexan cell cycle is still missing?

Are apicomplexan mitotic Crks (Crk4, Crk5, and Crk6) independent of cyclins? And if they are, what is the mechanism activating these kinases?

What factors evolved to fulfill the role of immediate effectors of the ‘new’ cell cycle Crks in apicomplexans?

## Glossary

Anaphase-promoting complex/cyclosome (APC/C): a multisubunit complex with E3 ligase activity that specifically targets mitotic cyclin B and securin for 26S proteasome degradation, leading to mitotic exit; the complex is regulated by the spindle assembly checkpoint control
ApiAP2: a family of apicomplexan proteins with a 60 amino acid DNA-binding domain related to APETALA transcription factors of plants
Bipartite centrosome: apicomplexan MTOC composed of two independently dividing cores with distinctive functions; the inner core is associated with chromosome segregation, and the outer core couples mitosis and cytokinesis during the budding cycle
Budding cycle: the last round of the polygenic cycle of apicomplexans (or the only division cycle of endodyogeny), in which segregation of duplicated chromosomes is synchronized with concerted cytokinesis
Cdk-related kinase (Crk): phylogenetically related to a Cdk kinase for which cognitive cyclin has not been identified
Centrocone: a localized mitotic structure first identified by TEM as an area of translucent material embedded in the nuclear envelope through which spindle microtubules connect kinetochores and the inner core of the bipartite centrosome
CEP proteins: coiled-coiled proteins of various sizes that locate to the centrosome (see centrosomeDB^i^)
Cyclin-dependent kinase (Cdk): a kinase for which there is experimental evidence for cyclin activation
E2F/DP-1: a heterodimer of two transcription factors that regulates G1-specific gene expression, and promotes G1/S transition
Endodyogeny: cell division that produces two internal daughter cells (called a tachyzoite in *T. gondii*)
Endopolygeny: a cell division that produces more than two internal daughter cells in a single cycle
Inner membrane complex (IMC): a cytoskeletal complex comprised of subpellicular microtubules over-layered with lattice proteins and flattened alveoli vesicles
Last eukaryotic common ancestor (LECA): a progenitor of all eukaryotes derived from prokaryotes by developing an endomembrane system about 800–1500 million years ago
Microtubule-organizing center (MTOC): a morphologically variable structure that nucleates microtubules for mitotic spindle apparatus, flagella/cilia and the subpellicular cytoskeleton in apicomplexan parasites
Nuclear cycle: a part of the polygenic cycle of apicomplexans where asynchronous rounds of chromosome replication and segregation are uncoupled from cytokinesis and G1 phase
Retinoblastoma protein (Rb): a pocket family protein that inhibits E2F/DP-1 activity, thus suppressing G1/S transition
Schizogony: a polygenic division where multiple daughter cells (called merozoites) bud from the mother cell plasmalemma
Semi-closed mitosis: in preparation for chromosome segregation, spindle microtubules are assembled from cytoplasmic tubulin subunits. Then, microtubules polymerize through local fenestra or clusters of nuclear pores. This mechanism differs from the open mitosis that occurs in the cytoplasm due to full disintegration of the nuclear membrane. It also differs from the closed mitosis where nuclear envelope remains fully intact
